# Cavity Ring-Down Spectroscopy Performance and Procedures for High-Throughput $\delta^{18}$ O and $\delta^{2}$ H Measurement in Water Using “Express” Mode

**DOI:** 10.1177/00037028241302355

**Published:** 2025-01-02

**Authors:** Nir Galili, Thomas M. Blattmann, Anna Somlyay, Nora Gallarotti, Timothy I. Eglinton, Jordon D. Hemingway

**Affiliations:** 1 Department of Earth Sciences, Geological Institute, 27219 ETH Zurich, Zurich, Switzerland

**Keywords:** Cavity ring-down spectroscopy, CRDS, δ^2^H, δ^18^O, spectroscopy, isotopic compositions, water analysis

## Abstract

Cavity ring-down spectroscopy (CRDS) is rapidly becoming an invaluable tool to measure hydrogen (δ²H) and oxygen (δ^18^O) isotopic compositions in water, yet the long-term accuracy and precision of this technique remain relatively underreported. Here, we critically evaluate one-year performance of CRDS δ²H and δ^18^O measurements at ETH Zurich, focusing on high throughput (~200 samples per week) while maintaining required precision and accuracy for diverse scientific investigations. We detail a comprehensive methodological and calibration strategy to optimize CRDS reliability for continuous, high-throughput analysis using Picarro’s “Express” mode, an area not extensively explored previously. Using this strategy, we demonstrate that CRDS achieves long-term precision better than ±0.5‰ for δ^18^O and ±1.0‰ for δ²H (±1σ) on three United States Geological Survey (USGS) reference materials treated as unknowns.^18^ Specifically, reported results for each reference material over this one-year period are: (i) USGS W-67444: $\delta^{2}$ H = −399.32±0.96‰ , $\delta^{18}$ O = −51.07±0.45‰ ( n=30 ), (ii) USGS W-67400: $\delta^{2}$ H = 2.55±0.49‰ , $\delta^{18}$ O = −1.85±0.13‰ ( n=140 ), and (iii) USGS-50: $\delta^{2}$ H = 33.68±0.91‰ , $\delta^{18}$ O = 5.03±0.04‰ ( n=21 ). We also address challenges such as aligning our analytical uncertainties with the narrower uncertainties of International Atomic Energy Agency reference materials, and mitigating inherent CRDS issues like memory and matrix effects when analyzing environmental samples. Our review provides a practical framework for CRDS applications in hydrology, paleoclimatology, and biogeochemistry, underscoring the importance of continuous evaluation and methodological refinement to ensure accuracy and precision in δ²H and δ^18^O analyses.^18^

## Graphical abstract

**Graphical abstract:**
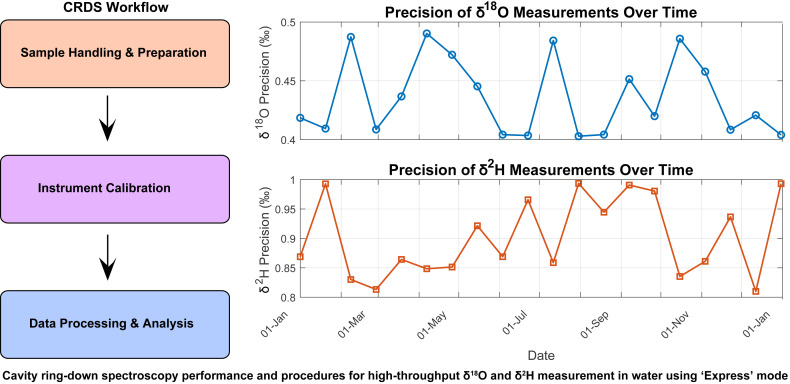
This is a visual representation of the abstract.

## Introduction

The stable hydrogen (^2^H/^1^H, reported as $\delta^{2}$ H) and oxygen (^18^O/^16^O, reported as $\delta^{18}$ O) isotopic compositions of H_2_O in its various phases—vapor, liquid, and solid—provide insight into hydrological budgets across spatiotemporal scales.^1–3^ Stable-isotope analysis is crucial for several hydrologic applications such as: constraining the origin of water bodies,^4^ quantifying water mixing processes,^2^ tracking precipitation source and amount,^5^ and tracing interactions between surface and subsurface water with biological ecosystems.^6^ Recently, significant analytical advances have been made regarding the stable-isotope measurement of H_2_O; beyond increased precision, these strides have prompted innovative applications in various Earth-science disciplines including hydrology, paleoclimatology, and biogeochemistry.^1,2,7,8^ Although triple oxygen isotopes (i.e., including ^17^O/^16^O in addition to ^18^O/^16^O) provide valuable additional information (e.g., Surma et al.^7^), their analysis requires specialized methods beyond the rapid CRDS techniques discussed here and is therefore outside the scope of this study.

Historically, the “state-of-the-art” for stable-isotope measurements has been isotope ratio mass spectrometry (IRMS).^9^ Despite the utility of this technique, sample throughput limitations and the complexity of sample preparation have underscored the need for more efficient methodologies. Over the past two decades, this demand has driven the progressive adoption of optical methods such as cavity ring-down spectroscopy (CRDS), which rely on the selective absorption of a laser beam.^10^ Recent studies have focused on balancing precision and throughput in isotope analysis using CRDS.^11–16^ However, despite this widespread adoption, several drawbacks related to CRDS memory effects and result accuracy remain,^10,13,17–19^ and independent (i.e., not relying on manufacturer white papers) long-term precision and accuracy evaluation of such systems is needed. Our study differs by providing a comprehensive, long-term evaluation of CRDS performance using the “Express” mode over a one-year period, offering new insights into its reliability and practical considerations for high-throughput analysis.

To evaluate such drawbacks while aiming to meet the requisite high-throughput, high-precision, and high-accuracy analyses, we conducted a year-long evaluation of CRDS performance at the Geological Institute, ETH Zurich. Despite achieving a conservative long-term precision better than ±0.5‰ for $\delta^{18}$ O and ±1.0‰ for $\delta^{2}$ H (all uncertainties reported here are ±1σ ), which aligns with the internationally recognized reference materials, we encountered notable challenges. These include aligning our results with established values for International Atomic Energy Agency (IAEA) reference materials and addressing systematic issues inherent to CRDS methodologies.^20^

This study details our methodological approach and calibration strategy, aiming to enhance the CRDS system’s reliability for continuous, high-throughput sample analysis. By analyzing three USGS reference materials as unknowns (alongside routine sample analyses) over a one-year period, we demonstrate our CRDS method’s capability to achieve benchmark precision and accuracy, thereby setting realistic expectations for its application in diverse scientific investigations. Our performance report serves as a guide for potential CRDS users, highlighting the system’s capabilities and limitations based on long-term empirical evidence and operational experience.

## Experimental Method

### Notation

Isotope ratios are represented relative to the international reference material Vienna Standard Mean Ocean Water (VSMOW) and expressed in traditional “delta” notation as

(1) $$\delta^{2}\text{H}=\frac{{}^{2}{\text{R}}_{\text{sample}}}{{}^{2}{\text{R}}_{\text{VSMOW}}}-1$$

and

(2) $$\delta^{18}\text{O}=\frac{{}^{18}{\text{R}}_{\text{sample}}}{{}^{18}{\text{R}}_{\text{VSMOW}}}-1\;$$

where ^2^R and ^18^R denote ^2^H/^1^H and ^18^O/^16^O ratios, respectively. Typically, $\delta^{2}$ H and $\delta^{18}$ O values are reported in units of “permil” ( ‰ ) by multiplying Eqs. 1 and 2 by 1000 .

### Instrumentation and Data/Acquisition

Stable hydrogen and oxygen isotopic compositions were analyzed using a Picarro high-precision vaporizer (Picarro, V1102-i; maintained at $110^{\circ }$ ), including a salt liner to protect against salt precipitation and minimize possible matrix effects, coupled to a CRDS isotope and gas concentration analyzer (L2130-i; using N_2_ as purge gas with background pressure set to 0.16 bar). The vaporizer syringe inlet was sealed with an SGE High-Temperature septum, which was replaced after each sequence to ensure the integrity of the seal of the vaporizer to the ambient atmosphere. Water samples were introduced using a pre-cleaned 10μ L SGE autosampler syringe with a fixed needle, and resulting signal levels ranged between 19 000 and 21 000 ppmv of water vapor. The Picarro CRDS software was run on version g2000-1.6.0.53(94c8f8e7) and the autosampler supplied by Picarro was a Moduvision Technologies BV ALS-G Autosampler.

**Figure 1.**
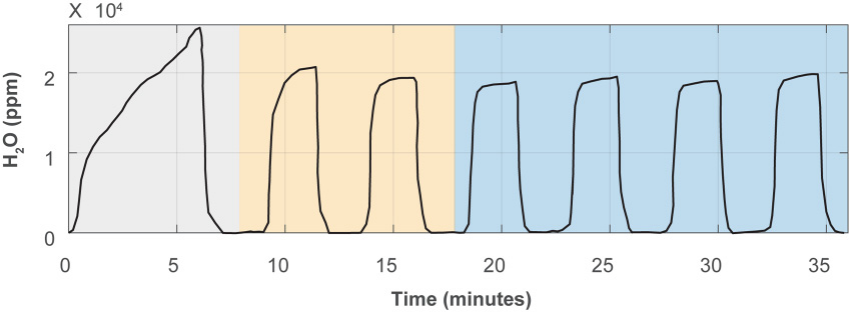
Injection sequence for Picarro’s “Express” mode. The mode allows the first six injections to be used to flush the vaporizer and CRDS measurement cavity between samples (single triangular pulse; gray-shaded region), followed by six injections for measurement introduced as individual pulses. In this study, the first two individual pulses after flushing (orange-shaded region) were omitted from data reduction whereas the final four injections were included in data reduction and analysis (blue-shaded region; see main text).

Each analysis comprised 12 injections of 2.1μ L each using the “Express” mode developed by Picarro. In this configuration, the first six injections are employed to flush the vaporizer and CRDS measurement cavity between samples and are not analyzed. This flushing step serves to remove memory effects and appears as a single triangular pulse on the water vapor trace measured by the CRDS cavity. The remaining six injections were introduced as individual pulses for measurement (Figure 1). For these six individual pulses, the first two were omitted from further data reduction as these frequently show anomalous peak shape and/or height related to the preceding purge procedure, which compromises measurement quality (e.g., first individual pulse in Figure 1). The final four pulses typically exhibit stable peak shape allowing for stable measurement performance and were thus employed for data reduction. The sample filling speed was set at 0.50μ Ls^−1^ with a post-fill delay of 2 s to allow relaxation of the vacuum gap. The sample injection speed into the vaporizer was set at 10μ L^−1^. Four rinses with the upcoming sample material of 2.50μ L each were performed prior to beginning the flushing. Between analyses, the syringe was cleaned with 5% orthophosphoric acid and Milli-Q water (18.2 M Ω · cm^−1^, <10 parts per billion (ppb) total organic carbon). To minimize waste and evaporative loss—particularly important for sample-limited reference materials—we utilized V-shaped 300μ L polypropylene vials with polytetrafluoroethylene-lined rubber septa, filled to 310μ L capacity to minimize evapotranspiration effects within the vials, and we centrifuged each vial prior to analysis to remove air bubbles. International reference materials, in-house standards, and samples were always analyzed within 96 h of V-shaped vial filling to ensure measurement integrity.

Isotopic compositions were calibrated against three IAEA reference materials: (i) Standard Light Antarctic Precipitation aliquot 2 (SLAP-2; consensus values $\delta^{2}$ H = −427.50±0.30‰ VSMOW, $\delta^{18}$ O = −55.50±0.02‰ VSMOW),^21^ (ii) Greenland Summit Precipitation (GRESP; consensus values $\delta^{2}$ H = −258.00±0.40‰ VSMOW, $\delta^{18}$ O = −33.40±0.04‰ VSMOW),^22^ and (iii) VSMOW aliquot 2 (VSMOW2; consensus values $\delta^{2}$ H = 0.00±0.30‰ , $\delta^{18}$ O = 0.00±0.30‰ VSMOW).^21^ Independent quality control was achieved by routinely analyzing three USGS reference materials as unknown samples: (i) USGS W-67444 (consensus values $\delta^{2}$ H = −399.10±0.50‰ VSMOW, $\delta^{18}$ O = −51.14±0.04‰ VSMOW),^23^ (ii) USGS W-67400 (consensus values $\delta^{2}$ H = 1.20±0.50‰ VSMOW, $\delta^{18}$ O = −1.97±0.04‰ VSMOW),^24^ and (iii) USGS-50 (consensus values $\delta^{2}$ H = 32.80±0.20‰ VSMOW, $\delta^{18}$ O = 4.95±0.01‰ VSMOW).^25^ We routinely utilized USGS W-67400 as the primary quality control, given its isotope composition falls within the typical environmental sample range. USGS W-67444 and USGS-50 were included only when analyzing isotopically enriched or depleted samples, respectively, to ensure accuracy across the range of expected sample values.

### Sampling Scheme

All analytical sessions employed a systematic standard measurement scheme (Table S1, Supplemental Material). Specifically, each session began with a Milli-Q water flush followed by three in-house standards analyzed in order from ^2^H- and ^18^O-depleted to enriched (Siberian water (SIBW), Lake Zurich water (ZW), Mediterranean Sea water; MEDW). These in-house standards are prepared through complete distillation to remove their salt content from the environmental water samples collected in their respective locations. All standards were analyzed from three separate vials, with the first vial of each standard (termed “pre”) comprising material utilized in a previous session. The purpose of the “pre” vial was to flush the vaporizer and CRDS cavity with a solution that approximates the actual value of the in-house standard to be measured, thereby minimizing deviations due to memory effect when cycling across a large isotope range. Results from “pre” vials were not used in data reduction. By utilizing previously measured aliquots, this practice additionally reduces standard material waste. Following in-house standards, three IAEA reference materials were analyzed, again in order from ^2^H- and ^18^O-depleted to enriched (SLAP-2, GRESP, and VSMOW-2). Similar to in-house standards, IAEA reference materials were analyzed from three separate vials, with the first vial of each comprising material utilized in a previous session (i.e., “pre” vials).

Sessions then proceeded with analyses of unknown samples. Typically, the first six sample vials contained USGS W-67400 (although some sessions contained more or fewer vials; see Statistical analysis section), which was treated identically to unknown samples. This reference material thus represents our primary independent quality control. Additional USGS reference materials (USGS-50 and USGS W-67444) were sometimes included depending on the expected sample isotopic composition range (again typically six vials, although some sessions contained more or fewer vials; see Statistical analysis section). When included, these materials were similarly treated identically to unknown samples. Finally, each session ended with the three in-house standards analyzed as described above; these served to bracket the entire sequence and monitor for instrument drift and matrix effects. Although typically only analyzed at the beginning and end of each session, one session additionally contained a mid-session set of in-house standards analyzed between unknown samples (3 July 2023; Tables S2–S4, Supplemental Material).

Following each session, data were reduced using a three-step procedure. In the first step, Picarro’s post-processing software was employed (ChemCorrect Viewer version 1.2.1.7 with supplier-provided instruction file “chemcorrect_inst_avg_orgeval_10.csv”). This software (i) reduces measurement injection pulses into averaged isotope compositions for each analysis, (ii) flags samples showing measurement anomalies (i.e., organic contamination as determined by spectral baseline slope and curvature), and (iii) calibrates results using user-inputted consensus values for reference material isotopic compositions. Here, calibrations were performed using ordinary least squares (OLS) regression results when including all analyses of the three in-house standards (i.e., three-point calibration with n=4 analyses per standard; n=6 for the analytical session of 3 July 2023).

The second step involved correcting ChemCorrect-calibrated data for any analytical drift using in-house standard results. Specifically, residuals were calculated as

(3) $$r_{i,j}=x_{i,j}-{\bar{x}}_{i},$$

where $x_{i,j}$ is the measured isotopic composition ( $\delta^{2}$ H or $\delta^{18}$ O) for analysis j of standard i and ${\bar{x}}_{i}$ is the session-averaged isotopic composition of standard i . Residuals for all analyses of all in-house standards were combined and regressed against sample number (i.e., proportional to time) using OLS. Regression slopes were then used to correct all reference material and sample analyses for instrument drift. However, drift-correction regression slopes were typically statistically insignificant ( p>0.05 ), resulting in no drift correction being applied.

Finally, in step three, drift-corrected IAEA reference material results were regressed against their consensus values using OLS, and regression results were used to further correct all measured unknown samples for any offsets, thus improving accuracy. For all sessions included here, IAEA standard regressions exhibited OLS slopes of 0.9856 to 0.9999 , intercepts of 0.55‰ to 0.80‰ for $\delta^{2}$ H and 1.45‰ to 1.60‰ for $\delta^{18}$ O, and regression fit ( $R^{2}$ values) >0.999 for both isotope systems. Regression results thus indicate high measurement precision after the first two data reduction steps, albeit with a statistically significant offset for $\delta^{18}$ O values.

### Statistical Analysis

Because USGS reference materials and our in-house standards were analyzed as unknown samples with differing numbers of analyses per session, here we report their results across multiple sessions as weighted means and standard deviations. Specifically, we calculate weighted means as

(4) $$\mu_{i}^{\text{m}}=\frac{\sum {\bar{x}}_{i,k}n_{i,k}}{\sum n_{i,k}}\;$$

where ${\bar{x}}_{i,k}$ is the session-averaged isotopic composition ( $\delta^{2}$ H or $\delta^{18}$ O) for session k of reference material or standard i , $n_{i,k}$ is the number of analyses for reference material or standard i in session k , and the superscript “m” indicates this calculation applies to measured values. We similarly calculate weighted standard deviations as

(5) $$\sigma_{i}^{\text{m}}=\sqrt{\frac{\sum n_{i,k}{\left(x_{i}-\mu_{i}\right)}^{2}}{\sum n_{i,k}}}$$

In contrast to USGS reference materials and our in-house standards, the number of analyses for IAEA reference materials ( n=2 ) was always identical across all sessions. For these materials, Eqs. 4 and 5 thus reduce to traditional (i.e., unweighted) means and standard deviations.

Following Wassenaar et al.,^26^ we determine USGS reference material measurement accuracy by calculating a z -score as

(6) $$z=\left|\frac{\mu_{i}^{\text{m}}-\mu_{i}^{\text{c}}}{\text{SPDA}}\right|\;$$

where superscript “c” indicates this calculation applies to consensus values such that $\mu_{i}^{\text{c}}$ is the consensus value of reference material i reported in the literature^23–25^ and SPDA is the “standardard deviation for proficiency assessment”, defined as 0.8‰ for $\delta^{2}$ H and 0.1‰ for $\delta^{18}$ O. Results are deemed “acceptable” if z<2.00 .^20^

Finally, we employ a Monte Carlo simulation technique to visualize measured and consensus USGS reference material distributions. Specifically, we assume all distributions are normally distributed such that measured and consensus random variables can be defined as

(7) $${\bar{x}}_{i,l}^{\text{m}}∼N(\mu_{i}^{\text{m}},\sigma_{i}^{\text{m}})$$

and

(8) $${\bar{x}}_{i,l}^{\text{c}}∼N(\mu_{i}^{\text{c}},\sigma_{i}^{\text{c}})$$

where ${\bar{x}}_{i,l}$ is the randomly drawn value of USGS reference material i for Monte Carlo iteration l , $\mu_{i}^{\text{m}}$ and $\sigma_{i}^{\text{m}}$ are calculated by Eqs. 4 and 5, and $\mu_{i}^{\text{c}}$ and $\sigma_{i}^{\text{c}}$ are reported in the literature.^23–25^ For all visualizations, we calculate ${\bar{x}}_{i,l}^{\text{m}}$ and ${\bar{x}}_{i,l}^{\text{c}}$ for n=100000 iterations, and we plot resulting synthetic distributions.

## Results and Discussion

### Long-Term Results and Comparison to Consensus Values

**Figure 2.**
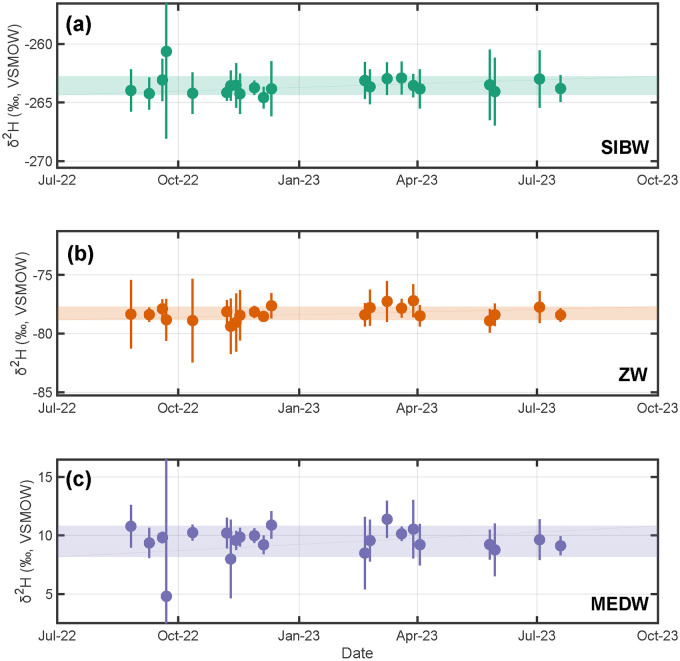
Measured stable-hydrogen isotopic compositions through time for our in-house standards (a) SIBW, (b) ZW, and (c) MEDW measured over a one-year period. Individual points and vertical bars are analytical session averages and ±1σ uncertainties. Horizontal shaded regions are weighted ±1σ uncertainties about the weighted mean of session averages (Eqs. 4 and 5; SIBW = −263.85±0.78‰ VSMOW; ZW = −78.26±0.55‰ VSMOW; MEDW = 9.50±1.27‰ VSMOW; n=90 analyses each).

**Figure 3.**
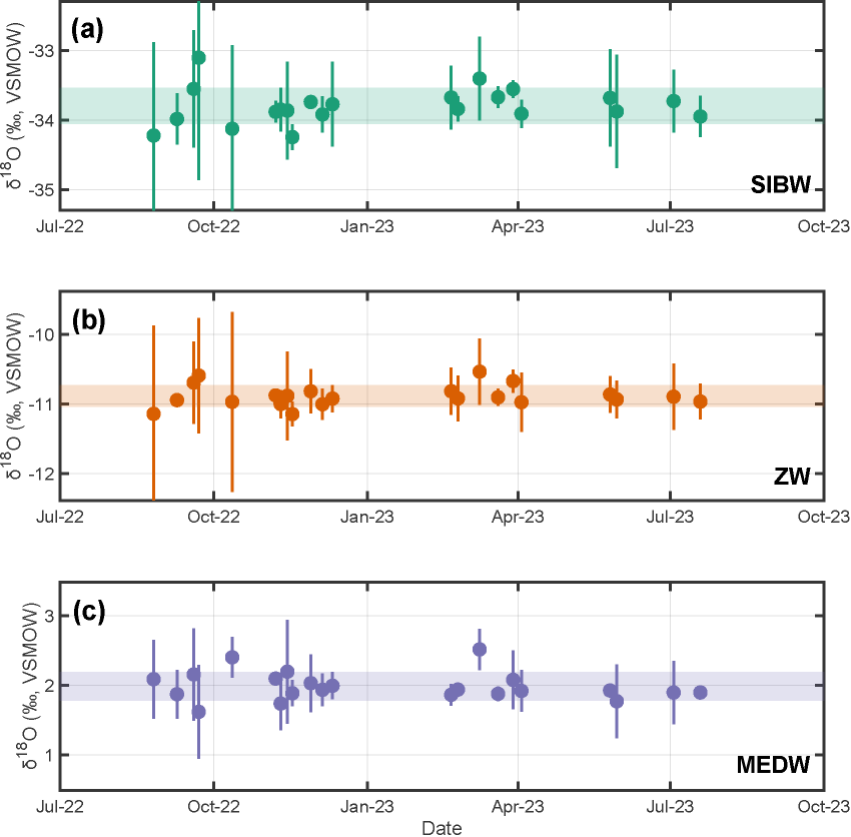
Measured stable-oxygen isotopic compositions through time for our in-house standards (a) SIBW, (b) ZW, and (c) MEDW measured over a one-year period. Individual points and vertical bars are analytical session averages and ±1σ uncertainties. Horizontal shaded regions are weighted ±1σ uncertainties about the weighted mean of session averages (Eqs. 4 and 5; SIBW = −33.80±0.25‰ VSMOW; ZW = −10.89±0.15‰ VSMOW; MEDW = 1.98±0.20‰ VSMOW; n=90 analyses each).

**Figure 4.**
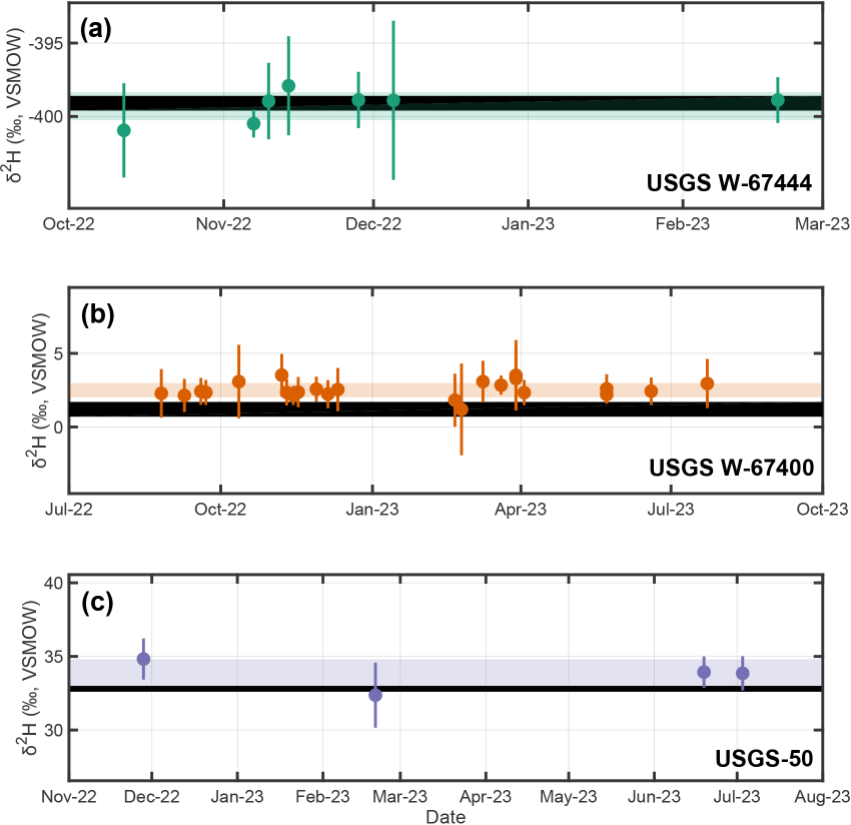
Consensus and measured stable-hydrogen isotopic compositions through time for USGS reference materials: (a) USGS W-67444, (b) USGS W-67400, and (c) USGS-50 measured over a one-year period. Individual points and vertical bars are analytical session averages and ±1σ uncertainties. Horizontal shaded regions are weighted ±1σ uncertainties about the weighted mean of session averages (Eqs. 4 and 5; USGS W-67444 = −399.32±0.96‰ VSMOW, n=30 analyses; USGS W-67400 = 2.55±0.49‰ VSMOW, n=140 analyses; USGS-50 = 32.80±0.20‰ VSMOW, n=21 analyses). Thick horizontal black lines are consensus ranges.^23–25^

**Figure 5.**
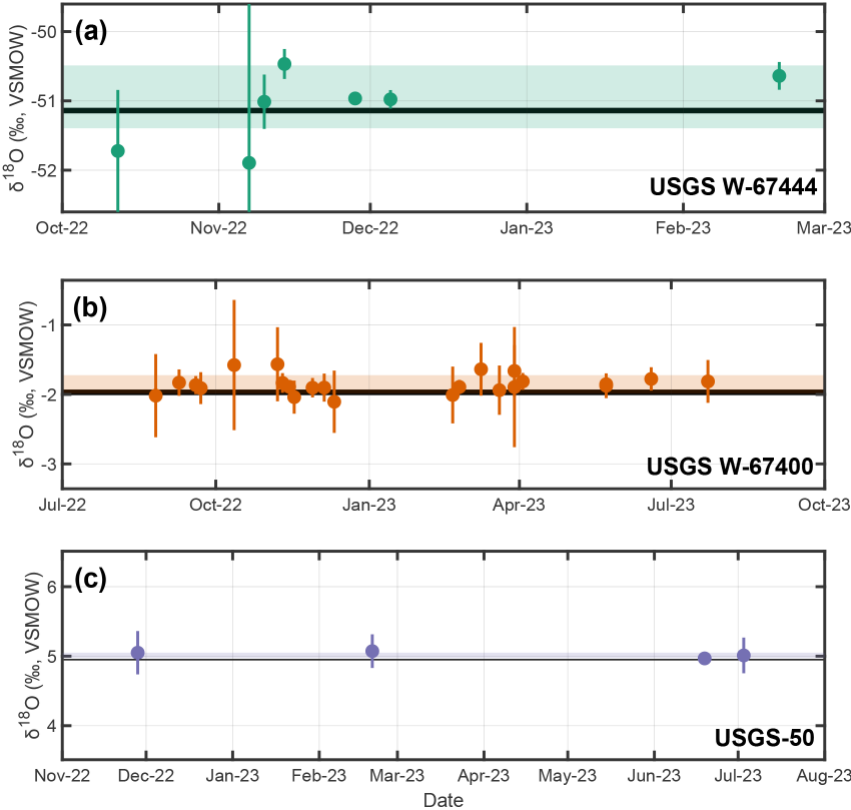
Consensus and measured stable-oxygen isotopic compositions through time for USGS reference materials: (a) USGS W-67444, (b) USGS W-67400, and (c) USGS-50 measured over a one-year period. Individual points and vertical bars are analytical session averages and ±1σ uncertainties. Horizontal shaded regions are weighted ±1σ uncertainties about the weighted mean of session averages (Eqs. 4 and 5; USGS W-67444 = −51.07±0.45‰ VSMOW, n=30 analyses; USGS W-67400 = −1.85±0.13‰ VSMOW, n=140 analyses; USGS-50 = 5.03±0.04‰ VSMOW, n=21 analyses). Thick horizontal black lines are consensus ranges.^23–25^

**Figure 6.**
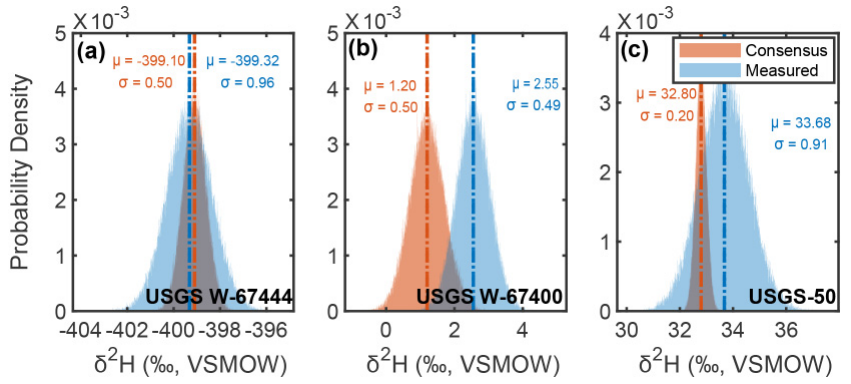
Comparison of consensus and long-term average measured stable-hydrogen isotopic composition distributions for USGS reference materials (a) USGS W-67444 ( z -score = 0.27), (b) USGS W-67400 ( z -score = 1.69), and (c) USGS-50 ( z -score = 1.10). Monte Carlo distributions are generated using Eqs. 7 and 8 using n=100000 iterations. All resulting z -scores indicate “acceptable” results following IAEA’s Water Isotope Inter-Comparison criteria,^20,26^ thus highlighting the ability of CRDS to capture hydrogen isotopic variations within water samples.

**Figure 7.**
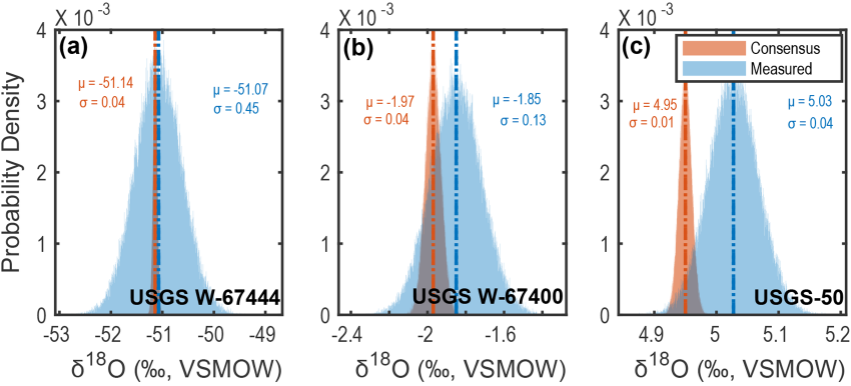
Comparison of consensus and long-term average measured stable-oxygen isotopic composition distributions for USGS reference materials: (a) USGS W-67444 ( z -score = 0.65), (b) USGS W-67400 ( z -score = 1.20), and (c) USGS-50 ( z -score = 0.78). Monte Carlo distributions are generated using Eqs. 7 and 8 using n=100000 iterations. All resulting z -scores indicate “acceptable” results following IAEA’s Water Isotope Inter-Comparison criteria,^20,26^ thus highlighting the ability of CRDS to capture hydrogen isotopic variations within water samples.

When corrected to IAEA reference material consensus values (i.e., during data reduction step three), resulting average isotopic compositions of our in-house standards measured over a one-year period are: (i) SIBW: $\delta^{2}$ H = −263.85±0.78‰ , $\delta^{18}$ O = −33.80±0.25‰ ( n=90 ; Table S2); (ii) ZW: $\delta^{2}$ H = −78.26±0.55‰ , $\delta^{18}$ O = −10.89±0.15‰ ( n=90 ; Table S3, Supplemental Material); and (iii) MEDW: $\delta^{2}$ H = 9.50±1.27‰ , $\delta^{18}$ O = 1.98±0.20‰ ( n=90 ; Table S4, Supplemental Material). We observe no statistically significant long-term shift in mean values or standard deviations as a function of time within this one-year period (Figures 2 and 3). Using these results and our three-step data reduction procedure, we additionally report long-term isotopic compositions for USGS reference materials analyzed as unknown samples. These are: (i) USGS W-67444: $\delta^{2}$ H = −399.32±0.96‰ , $\delta^{18}$ O = −51.07±0.45‰ ( n=30 ; Table S5, Supplemental Material); (ii) USGS W-67400: $\delta^{2}$ H = 2.55±0.49‰ , $\delta^{18}$ O = −1.85±0.13‰ ( n=140 ; Table S6, Supplemental Material); and (iii) USGS-50: $\delta^{2}$ H = 33.68±0.91‰ , $\delta^{18}$ O = 5.03±0.04‰ ( n=21 ; Table S7, Supplemental Material). Like for in-house standards, we observe no statistically significant long-term shift in mean values or standard deviations as a function of time within this one-year period (Figures 4 and 5), although measured standard deviations calculated here are typically larger than reported consensus uncertainty (Figures 6 and 7). Additionally, the consistently higher values for USGS W-67400 may indicate a calibration shift, likely due to its proximity to the bracketed IAEA VSMOW reference material. This suggests the potential benefit of developing additional reference materials with heavier values within the community. To address concerns about potential evaporation from the V-shaped 300μ L polypropylene vials used during analysis, we conducted a long-term test where USGS W-67400 reference water was stored under room temperature conditions. The reference water storage began on 24 March 2023 and was measured on 3 July 2023, yielding $\delta^{18}$ O = −1.52±0.20‰ and $\delta^{2}$ H = 4.02±0.61‰ ( n=6 ). It was measured again on 22 October 2023, yielding $\delta^{18}$ O = −1.06±0.26‰ and $\delta^{2}$ H = 5.30±0.92‰ ( n=12 ). Compared to the consensus values of $\delta^{18}$ O = −1.97±0.02‰ ( ±1σ ) and $\delta^{2}$ H = 1.20±0.50‰ ( ±1σ ), there is a significant change toward higher (heavier) values over timescales of weeks. This demonstrates that our measurement timescale ( <1 week) is acceptable. To mitigate the effects of septa perforations, we use Teflon-lined rubber septa, known for their high-quality resealing properties.^27^

Following the IAEA’s Water Isotope Inter-Comparison criteria,^20,26^ all values measured here are consistent with consensus values reported by the USGS^23–25^ (i.e., z<2.00 , indicating “acceptable”). Specifically, z≤1.69 for all of our USGS reference material results, ranging from a minimum of z=0.27 for USGS W-67444 $\delta^{2}$ H values and a maximum of z=1.69 for USGS W-67400 $\delta^{2}$ H values (average z=1.0 for both $\delta^{2}$ H and $\delta^{18}$ O values; Figures 6 and 7). These results can be interpreted within the context of an existing inter-laboratory evaluation using average z -scores for a core set of six calibration standards.^20,26^ When compared to available data from^26^ for all participating laboratories ( n=287 ), values reported here are in the top 35^th^ percentiles for both $\delta^{2}$ H and $\delta^{18}$ O, highlighting the robustness of our analytical and data-reduction approach. Despite such accurate overall results, we do observe differences in precision between USGS reference materials. For example, the higher z -score for USGS W-67444 $\delta^{2}$ H values suggests the need for further investigation into potential limitations of the CRDS technology, calibration nuances, or the impact of memory effects.

### Mitigating Memory and Matrix Effects

Historically, memory effects have posed a significant challenge to measuring reliable isotopic compositions using CRDS.^10,19,28^ Such effects are known to introduce errors that lower precision and accuracy, and several methods have been developed to mitigate them. For example, previous work has suggested employing several injections to remove memory effects^19^ or applying asymptotic mathematical approaches^28^ to extrapolate and estimate true sample values. Our protocol aims to minimize $\delta^{2}$ H and $\delta^{18}$ O memory effects in two ways: (i) by utilizing Picarro’s “Express” mode, in which the first six injections of each analysis are used to flush the vaporizer and CRDS measurement cavity, and (ii) by omitting the first two injection pulses after flushing, which often display anomalous peak shapes (Figure 1). In this study, we chose six injections to balance performance and throughput. Increasing the number of injections could improve precision but would reduce the number of samples that can be analyzed within a given time. Nevertheless, several studies have highlighted how residual signals from previous samples can subtly influence subsequent measurements, particularly in high-throughput analyses with a broad isotopic range, or when the triple oxygen isotope composition of water is of interest.^13,10,17–19^

**Figure 8.**
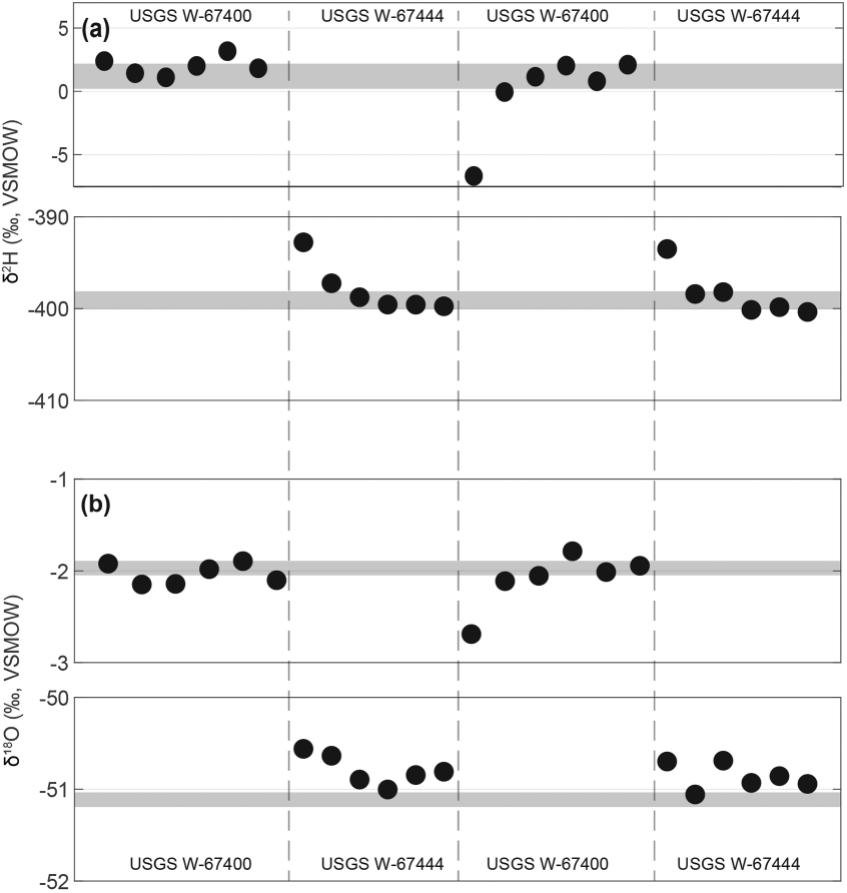
Demonstrating the memory effect for (a) stable-hydrogen and (b) stable-oxygen measurements when alternating measurements between reference materials with largely contrasting isotopic compositions (USGS W-67400 and USGS W-67444). Black circles are results for individual pulses within a given analysis, whereas horizontal gray-shaded regions are consensus ranges.^23,24^ For all injections, memory effects become statistically insignificant after the six flushes and two individual pulses, supporting the omission of these pulses during data reduction in our protocol (cf. Figure 1).

To test this effect, we employed an experimental sequence in which USGS W-67400 and USGS W-67444 reference materials—whose consensus $\delta^{2}$ H and $\delta^{18}$ O values differ by 400.30±0.71‰ and 49.13±0.06‰ , respectively—were analyzed in alternating fashion. Such large isotopic juxtaposition allows us to assess carryover memory effects between samples in our instrumental setup, particularly when using Picarro’s “Express” mode. As shown in Figure 8, the first two injection pulses after flushing can be impacted by memory effects, but any drift becomes statistically insignificant by the third injection, even when alternating between samples with such large isotopic differences. This result supports our decision to omit the first two injections after flushing from subsequent data reduction and emphasizes that memory effects should be negligible when following the protocol developed here. Still, in cases where large isotopic differences are expected between samples, a “pre” vial with similar isotopic composition to subsequent samples can be analyzed as a precaution. In the Geological Institute at ETH Zurich, such “pre” vials are typically analyzed when switching between sample batches of different origins on the same sequence and are always analyzed prior to all in-house standards and IAEA reference materials (Table S1, Supplemental Material).

Beyond memory effects, our long-term quality-control assessment also allows us to assess the importance of any so-called “matrix” effects due to salt accumulation and carryover. Such matrix effects are known to impact precision, drift, and memory in laser-based spectroscopy analyzer systems, particularly when processing environmental samples.^29^ To assess matrix effects, here we compare calculated analytical uncertainty between USGS reference materials—which are only analyzed at the beginning of an analytical session, just after flushing and prior to natural samples (and in-house standards), which are analyzed both before and after natural samples (Table S1, Supplemental Material). Thus, if matrix effects are important, we expect larger analytical uncertainty for in-house standards relative to USGS reference materials, particularly for analyses performed after natural samples.

Our analyses reveal no statistically significant differences in the long-term analytical uncertainties for both $\delta^{18}$ O and $\delta^{2}$ H between USGS reference materials and our in-house standards when including all analyses (two-tailed t -test, p -values = 0.61 and 0.43, respectively). Matrix effects therefore do not appear to substantially compromise the precision or accuracy of our measurements over an entire analytical session. However, when separating in-house standard results into those before and after natural samples, we observe a statistically significant increase in analytical uncertainty for both $\delta^{18}$ O and $\delta^{2}$ H for post-natural sample analyses (uncertainty defined as the absolute difference between the maximum and minimum measurement values; two-tailed t -test, p -values <0.05 ; Figure S1, Supplemental Material). This observation underscores the necessity of robust flushing and maintenance protocols, particularly when handling environmental samples that may exacerbate salt accumulation and other matrix-related interferences. Nevertheless, the observation that long-term precisions of USGS reference materials and in-house standards are statistically identical when including all pre- and post-natural sample analyses suggests that our protocol effectively achieves high-throughput performance while minimizing the effects of matrix interferences when working with environmental samples.

### Temporal Trends

We assessed potential temporal trends both for USGS reference materials and our in-house standards by regressing analytical session mean values as a function of time using OLS. Results indicate no statistically significant temporal trends for either $\delta^{18}$ O or $\delta^{2}$ H across all reference materials ( p -value > 0.05 always; Figures 2–5). Specifically, $\delta^{18}$ O slopes ranged from a minimum of −0.00034‰ day^−1^ for USGS-50 to a maximum of 0.00732‰ day^−1^ for USGS W-67444, with neither trend being statistically significant ( p values = 0.24 and 0.18, respectively). Similarly, $\delta^{2}$ H slopes were statistically insignificant and ranged from a minimum of −0.00168‰ day^−1^ for USGS-50 to a maximum of 0.01117‰ day^−1^ for USGS W-67444 ( p values = 0.83 and 0.33, respectively). Such a lack of significant temporal trends affirm the long-term stability of CRDS measurement systems.

## Conclusion

In this study, we evaluated the performance of CRDS for the measurement of $\delta^{2}$ H and $\delta^{18}$ O in water samples over a one-year period at the Geological Institute, ETH Zurich. Our analysis, grounded in the use of USGS reference materials analyzed as unknowns and tested against IAEA reference materials, establishes a baseline for the precision and accuracy attainable with CRDS, particularly when utilizing Picarro’s “Express” mode. Although CRDS has been promoted for its advantages in sample throughput and ease of use for water isotope analysis,^30^ our findings suggest that it does not reach the precision levels reported by the reference material consensus values^21–25^ (cf. Wassenaar et al.^30^). Despite this, the higher throughput capability of CRDS presents it as a valuable tool for projects requiring quick analysis of large sample sets, as well as those that can accept a slight decrease in precision and accuracy relative to IRMS. Our exploration of memory and matrix effects, known challenges in CRDS analyses,^10,19,28^ confirms that their impact can be effectively managed with existing instrumental capabilities. In particular, Picarro’s “Express” mode offers a pragmatic approach to mitigating memory effects without the need for extensive methodological innovations. These findings highlight the importance of careful calibration strategies and suggest that achieving the precision and accuracy specified by the manufacturer may vary across different instruments. This can be addressed by using a wider range of reference materials to cover the expected isotopic range of samples, as well as implementing advanced flushing protocols to reduce memory effects and minimize matrix influences when analyzing environmental samples. Additionally, adopting statistical techniques such as Monte Carlo simulations to assess measured versus consensus value precisions could enhance the interpretation of discrepancies, offering insights into their statistical significance and the reliability of CRDS measurements.

## Supplemental Material

sj-pdf-1-asp-10.1177_00037028241302355 - Supplemental material for Cavity Ring-Down Spectroscopy Performance and Procedures for High-Throughput $\delta^{18}$ O and $\delta^{2}$ H Measurement in Water Using “Express” Mode Supplemental material, sj-pdf-1-asp-10.1177_00037028241302355 for Cavity Ring-Down Spectroscopy Performance and Procedures for High-Throughput $\delta^{18}$ O and $\delta^{2}$ H Measurement in Water Using “Express” Mode by Nir Galili, Thomas M. Blattmann, Anna Somlyay, Nora Gallarotti, Timothy I. Eglinton, and Jordon D. Hemingway in Applied Spectroscopy
