# Supplementary material for: Cavity Ring-Down Spectroscopy Performance and Procedures for High-Throughput δ18 O and δ2 H Measurement in Water Using “Express” Mode
Source: Appl Spectrosc. 2025 Jan 2;79(7):1120–8. doi: 10.1177/00037028241302355 (PMC12231800; doi:10.1177/00037028241302355)
Supplement: sj-pdf-1-asp-10.1177_00037028241302355 - Supplemental material for Cavity Ring-Down Spectroscopy Performance and Procedures for High-Throughput δ18 O and δ2 H Measurement in Water Using “Express” Mode [file sj-pdf-1-asp-10.1177_00037028241302355.pdf]

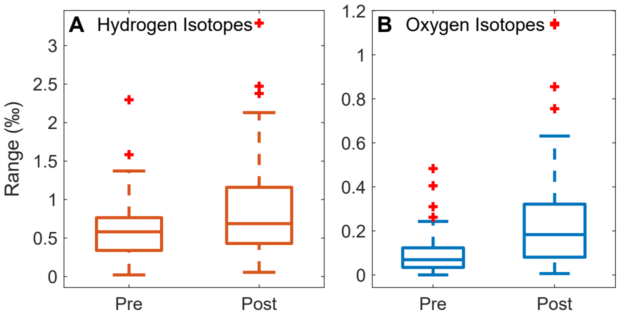

Figure S1. In-house standard uncertainties for analyses measured before ("Pre") and after ("Post") natural samples within a given sequence, separated into (A) stable-hydrogen and (B) stable-oxygen isotopic compositions (note that mid-session standards from 03.07.2023 are not included here). Because each sequence includes $n = 2$ analyses of each standard each for "Pre" and "Post" conditions, uncertainties are reported here as the absolute range between these duplicates, and ranges for all three standards are combined into a single box-and-whisker plot. For both isotopic systems, "Post" uncertainties are statistically greater than "Pre" uncertainties (two-tailed t-test, p-values < 0.05). These results underscore the impact of environmental sample processing on measurement reliability and highlight the importance of robust protocols to mitigate matrix effects.

Table S1. Order of vials used for a typical analytical session.

Vial	Identifier	Description
1	MQ-W	Warm-up
2	SIBWpre	Memory clean-up
3-4	SIBW	In-house standard with low δ^2H and δ^{18}O
5	ZWpre	Memory clean-up
6-7	ZW	In-house standard with intermediate δ^2H and δ^{18}O
8	MEDWpre	Memory clean-up
9-10	MEDW	In-house standard with high δ^2H and δ^{18}O
11	SLAP2pre	Memory clean-up
12-13	SLAP2	IAEA reference material with low δ^2H and δ^{18}O
14	GRESPpre	Memory clean-up
15-16	GRESP	IAEA reference material with intermediate δ^2H and δ^{18}O
17	VSMOW2pre	Memory clean-up
18-19	VSMOW2	IAEA reference material with high δ^2H and δ^{18}O
20-25	W-67400	USGS reference material (analyzed as unknown)
26-96	Samples	Up to 71 samples
97	SIBWpre	Memory clean-up
98-99	SIBW	In-house standard with low δ^2H and δ^{18}O
100	ZWpre	Memory clean-up
101-102	ZW	In-house standard with intermediate δ^2H and δ^{18}O
103	MEDWpre	Memory clean-up
104-105	MEDW	In-house standard with high δ^2H and δ^{18}O

Table S2. Long-term experimental results of Siberian water in-house standard (SIBW). Isotopic values are reported on the VSMOW-SLAP scale, with uncertainties expressed as $\pm 1\sigma$.

δ^2H	δ^{18}O	n	Date
-263.55 ± 0.78	-33.80 ± 0.25	All (90)	-
-263.97 ± 0.89	-34.22 ± 0.67	4	26.08.2022
-264.23 ± 0.69	-33.98 ± 0.18	4	09.09.2022
-263.08 ± 0.90	-33.55 ± 0.42	4	19.09.2022
-260.64 ± 3.72	-33.11 ± 0.88	4	22.09.2022
-264.21 ± 0.88	-34.13 ± 0.60	4	12.10.2022
-264.14 ± 0.34	-33.88 ± 0.08	4	07.11.2022
-263.56 ± 0.65	-33.85 ± 0.16	4	10.11.2022
-263.53 ± 0.94	-33.86 ± 0.35	4	14.11.2022
-264.25 ± 0.85	-34.24 ± 0.09	4	17.11.2022
-263.73 ± 0.31	-33.74 ± 0.02	4	28.11.2022
-264.57 ± 0.47	-33.92 ± 0.13	4	05.12.2022
-263.82 ± 1.17	-33.77 ± 0.30	4	11.12.2022
-263.66 ± 0.74	-33.84 ± 0.09	4	24.02.2023
-263.12 ± 0.78	-33.68 ± 0.23	4	20.02.2023
-262.96 ± 0.70	-33.40 ± 0.30	4	09.03.2023
-262.91 ± 0.70	-33.67 ± 0.08	4	20.03.2023
-263.55 ± 0.50	-33.55 ± 0.06	4	29.03.2023
-263.84 ± 0.84	-33.91 ± 0.10	4	03.04.2023
-263.48 ± 1.50	-33.68 ± 0.35	4	26.05.2023
-264.06 ± 1.44	-33.88 ± 0.41	4	30.05.2023
-263.80 ± 0.57	-33.95 ± 0.15	4	19.07.2023
-262.99 ± 1.22	-33.73 ± 0.22	6	03.07.2023

Table S3. Long-term experimental results of Lake Zurich water in-house standard (ZW). Isotopic values are reported on the VSMOW-SLAP scale, with uncertainties expressed as $\pm 1\sigma$.

δ^{2}H	δ^{18}O	n	Date
-78.27 ± 0.55	-10.89 ± 0.15	All (90)	-
-78.34 ± 1.46	-11.14 ± 0.63	4	26.08.2022
-78.38 ± 0.31	-10.94 ± 0.04	4	09.09.2022
-77.89 ± 0.40	-10.69 ± 0.30	4	19.09.2022
-78.82 ± 0.89	-10.59 ± 0.41	4	22.09.2022
-78.89 ± 1.78	-10.97 ± 0.65	4	12.10.2022
-78.12 ± 0.50	-10.88 ± 0.04	4	07.11.2022
-79.38 ± 1.18	-11.00 ± 0.10	4	10.11.2022
-79.05 ± 1.24	-10.88 ± 0.32	4	14.11.2022
-78.43 ± 1.07	-11.15 ± 0.09	4	17.11.2022
-78.14 ± 0.26	-10.82 ± 0.16	4	28.11.2022
-78.54 ± 0.22	-11.00 ± 0.11	4	05.12.2022
-77.62 ± 0.53	-10.92 ± 0.10	4	11.12.2022
-77.79 ± 0.77	-10.92 ± 0.16	4	24.02.2023
-78.40 ± 0.50	-10.82 ± 0.17	4	20.02.2023
-77.25 ± 0.86	-10.54 ± 0.24	4	09.03.2023
-77.83 ± 0.40	-10.90 ± 0.06	4	20.03.2023
-77.20 ± 0.70	-10.67 ± 0.08	4	29.03.2023
-78.48 ± 0.46	-10.97 ± 0.21	4	03.04.2023
-78.91 ± 0.50	-10.86 ± 0.13	4	26.05.2023
-78.39 ± 0.47	-10.93 ± 0.14	4	30.05.2023
-78.41 ± 0.29	-10.97 ± 0.13	4	19.07.2023
-77.74 ± 0.67	-10.90 ± 0.24	6	03.07.2023

Table S4. Long-term experimental results of Mediterranean Sea water in-house standard (MEDW). Isotopic values are reported on the VSMOW-SLAP scale, with uncertainties expressed as $\pm 1\sigma$.

δ^2H	δ^{18}O	n	Date
9.49 ± 1.27	1.99 ± 0.20	All (90)	-
10.79 ± 0.91	2.08 ± 0.28	4	26.08.2022
9.36 ± 0.64	1.87 ± 0.17	4	09.09.2022
9.83 ± 0.25	2.16 ± 0.33	4	19.09.2022
4.80 ± 5.92	1.62 ± 0.34	4	22.09.2022
10.25 ± 0.34	2.40 ± 0.15	4	12.10.2022
10.21 ± 0.66	2.10 ± 0.04	4	07.11.2022
7.99 ± 1.67	1.74 ± 0.19	4	10.11.2022
9.59 ± 0.40	2.20 ± 0.37	4	14.11.2022
9.86 ± 0.39	1.89 ± 0.09	4	17.11.2022
9.99 ± 0.32	2.03 ± 0.21	4	28.11.2022
9.20 ± 0.39	1.94 ± 0.12	4	05.12.2022
10.89 ± 0.59	1.99 ± 0.10	4	11.12.2022
9.56 ± 0.89	1.94 ± 0.04	4	24.02.2023
8.50 ± 1.54	1.86 ± 0.08	4	20.02.2023
11.39 ± 0.79	2.52 ± 0.15	4	09.03.2023
10.14 ± 0.30	1.88 ± 0.05	4	20.03.2023
10.54 ± 1.24	2.08 ± 0.21	4	29.03.2023
9.21 ± 0.88	1.92 ± 0.15	4	03.04.2023
9.23 ± 0.64	1.93 ± 0.04	4	26.05.2023
8.77 ± 1.13	1.77 ± 0.26	4	30.05.2023
9.12 ± 0.40	1.90 ± 0.05	4	19.07.2023
9.64 ± 0.86	1.90 ± 0.23	6	03.07.2023

Table S5. Long-term experimental results of USGS W-67444 reference material. Isotopic values are reported on the VSMOW-SLAP scale, with uncertainties expressed as $\pm 1\sigma$.

δ^2H	δ^{18}O	n	Date
-399.10 ± 0.50	-51.14 ± 0.04	Consensus*	-
-399.32 ± 0.96	-51.08 ± 0.45	All (30)	-
-400.95 ± 1.61	-51.72 ± 0.44	6	12.10.2022
-400.49 ± 0.47	-51.89 ± 1.31	2	07.11.2022
-398.95 ± 1.30	-51.01 ± 0.20	6	10.11.2022
-397.91 ± 1.69	-50.47 ± 0.11	3	14.11.2022
-398.88 ± 0.96	-50.97 ± 0.04	4	28.11.2022
-398.90 ± 2.71	-50.98 ± 0.07	3	05.12.2022
-398.88 ± 0.78	-50.64 ± 0.10	6	20.02.2023

*Consensus values from (23)

Table S6. Long-term experimental results of USGS W-67400 reference material. Isotopic values are reported on the VSMOW-SLAP scale, with uncertainties expressed as $\pm 1\sigma$.

δ^2H	δ^{18}O	n	Date
1.20 ± 0.50	-1.97 ± 0.04	Consensus*	-
2.55 ± 0.49	-1.85 ± 0.13	All (140)	-
2.28 ± 0.83	-2.02 ± 0.30	6	26.08.2022
2.14 ± 0.56	-1.83 ± 0.09	6	09.09.2022
2.42 ± 0.46	-1.87 ± 0.07	6	19.09.2022
2.34 ± 0.43	-1.91 ± 0.11	6	22.09.2022
3.08 ± 1.25	-1.58 ± 0.47	6	12.10.2022
3.52 ± 0.72	-1.57 ± 0.27	6	07.11.2022
2.35 ± 0.44	-1.83 ± 0.07	6	10.11.2022
2.14 ± 0.33	-1.89 ± 0.03	6	14.11.2022
2.37 ± 0.51	-2.04 ± 0.12	6	17.11.2022
2.57 ± 0.42	-1.90 ± 0.07	6	28.11.2022
2.23 ± 0.48	-1.90 ± 0.10	6	05.12.2022
2.54 ± 0.73	-2.11 ± 0.22	6	11.12.2022
1.19 ± 1.56	-1.89 ± 0.05	4	24.02.2023
1.81 ± 0.90	-2.01 ± 0.20	5	20.02.2023
3.08 ± 0.71	-1.64 ± 0.19	6	09.03.2023
2.84 ± 0.33	-1.94 ± 0.18	6	20.03.2023
3.29 ± 1.05	-1.66 ± 0.18	5	29.03.2023
3.51 ± 1.20	-1.89 ± 0.43	4	03.04.2023
2.34 ± 0.43	-1.81 ± 0.06	10	03.04.2023
2.20 ± 0.31	-1.85 ± 0.02	6	23.05.2023
2.59 ± 0.49	-1.88 ± 0.09	6	23.05.2023
2.43 ± 0.46	-1.78 ± 0.08	4	19.06.2023
2.95 ± 0.84	-1.81 ± 0.15	10	23.07.2023

*Consensus values from (24)

Table S7. Long-term experimental results of USGS-50 reference material. Isotopic values are reported on the VSMOW-SLAP scale, with uncertainties expressed as $\pm 1\sigma$.

δ^2H	δ^{18}O	n	Date
32.80 ± 0.20	4.95 ± 0.01	Consensus*	-
33.68 ± 0.91	5.03 ± 0.04	All (21)	-
34.82 ± 0.70	5.05 ± 0.16	5	28.11.2022
32.37 ± 1.10	5.07 ± 0.12	6	20.02.2023
33.94 ± 0.53	4.97 ± 0.05	5	19.06.2023
33.85 ± 0.58	5.01 ± 0.13	5	03.07.2023

*Consensus values from (25)
